# Efficacy of probiotic supplementation in reducing primary dysmenorrhea: a double-blinded randomized controlled trial

**DOI:** 10.1038/s41598-026-44327-5

**Published:** 2026-03-17

**Authors:** Orawin Vallibhakara, Waritsara Tosiri, Sakda Arj-Ong Vallibhakara, Areepan Sophonsritsuk, Nantaporn Lekpittaya

**Affiliations:** 1 Menopause Unit, Reproductive Endocrinology and Infertility Division, Department of Obstetrics and Gynaecology, Faculty of Medicine Ramathibodi Hospital, Mahidol University, Bangkok, Thailand; 2 Department of Obstetrics and Gynaecology, Faculty of Medicine Ramathibodi Hospital, Mahidol University, Bangkok, Thailand; 3 Interdisciplinary Studies and Lifelong Education (I-GRAD), Faculty of Public Health, Mahidol University, Bangkok, Thailand; 4 Reproductive Endocrinology and Infertility Unit, Department of Obstetrics and Gynaecology, Faculty of Medicine, Ramathibodi Hospital, Mahidol University, Bangkok, Thailand; 5 Clinical Pharmacy Section, Faculty of Medicine, Ramathibodi Hospital, Mahidol University, Bangkok, Thailand

**Keywords:** Probiotics, Primary dysmenorrhea, Menstrual pain, Complementary therapy, Randomized controlled trial, Diseases, Health care, Medical research

## Abstract

Primary dysmenorrhea is painful menstruation in the absence of pelvic pathology, whereas secondary dysmenorrhea is menstrual pain attributable to an underlying pelvic disease (e.g., endometriosis). Nonsteroidal anti-inflammatory drugs (NSAIDs) are commonly used as first-line therapy. Recent evidence ^18^ suggests that modulation of the gut microbiota may influence menstrual pain through immunologic and neuroendocrine pathways. This double-blinded, randomized, placebo-controlled trial investigated the efficacy of daily multispecies probiotic supplementation in reducing menstrual pain severity in women aged 18–24 years diagnosed with moderate to severe primary dysmenorrhea. Forty-eight participants were randomized to receive either a probiotic supplement or placebo for three consecutive menstrual cycles, followed by a three-month observation period. The primary outcome was the change in pain severity, assessed using a 10 cm visual analog scale (VAS). At baseline, VAS scores were similar between groups (probiotic 6.1 ± 1.17 vs placebo 6.3 ± 1.26; p = 0.62). After three months of intervention, the probiotic group demonstrated a significantly greater reduction in pain scores compared to the placebo group (3.7 ± 1.84 vs 5.8 ± 2.14; p < 0.01). However, the effect was not sustained after discontinuation of supplementation. No serious adverse events were reported. These findings suggest that continuous intake of multispecies probiotics may be an effective non-hormonal adjunct therapy for primary dysmenorrhea. Clinical trial registration: Thai Clinical Trials Registry (TCTR20230326001), registered on 22 March 2023. https://thaiclinicaltrials.org/

## Introduction

Dysmenorrhea, or menstrual pain, refers to cramping or abdominal discomfort accompanying menstruation. It is broadly classified as primary and secondary dysmenorrhea. Primary dysmenorrhea is painful menstruation in the absence of identifiable pelvic pathology and is most prevalent among adolescents and young women, whereas secondary dysmenorrhea is menstrual pain associated with underlying pelvic conditions such as endometriosis or adenomyosis^1,2^. Typically presenting in the lower abdomen and radiating to the back or thighs, the pain often coincides with the onset of menstrual bleeding and may persist for up to 72 h^1^. Additional symptoms, such as nausea, fatigue, headache, and sleep disturbances, frequently occur, contributing to a reduced quality of life and impaired daily functioning. Dysmenorrhea has been associated with school and work absenteeism, decreased productivity, and mental health challenges, including depression and anxiety^3,4^. Despite being the most prevalent gynecological condition among women of reproductive age, with an estimated prevalence ranging from 45 to 95%, dysmenorrhea remains underdiagnosed and undertreated^5–8^. Many individuals perceive menstrual pain as a normal part of menstruation and therefore rely on self-care or over-the-counter medications rather than seeking medical evaluation; limited access to healthcare, concerns about treatment effectiveness, and preferences regarding available treatment options may further contribute to underuse of evidence-based management^2^.

Although uterine prostaglandin overproduction—particularly prostaglandin F2α (PGF2α) and PGE2—is well established as a key pathophysiological mechanism, increasing evidence suggests that systemic and local inflammatory responses also play a role in the etiology of this condition. Elevated levels of pro-inflammatory cytokines, such as interleukin (IL)−6, tumor necrosis factor-alpha (TNF-α), and IL-1β, have been detected during the luteal and menstrual phases in women with primary dysmenorrhea, suggesting an aberrant immune-inflammatory state that extends beyond local prostaglandin synthesis. These inflammatory mediators may amplify nociceptive signaling and contribute to heightened pain sensitivity during menstruation^9,10^. Standard treatment for primary dysmenorrhea includes nonsteroidal anti-inflammatory drugs (NSAIDs) and hormonal contraceptives^11,12^^.^ However, interest in complementary and alternative therapies—such as acupuncture, herbal medicine, and yoga—has grown due to patient preference for non-pharmacological options^13–15^.

Probiotics have emerged as promising immunomodulatory agents capable of restoring microbial balance and attenuating inflammatory responses. Through modulation of gut microbiota composition, enhancement of mucosal barrier integrity, and regulation of cytokine profiles, probiotics—particularly Lactobacillus and Bifidobacterium species—have demonstrated systemic anti-inflammatory effects in various conditions, including metabolic syndrome, type 2 diabetes, and polycystic ovary syndrome^16–18^. Moreover, probiotics influence the gut–brain–immune axis and may affect estrogen metabolism via the estrobolome, thereby potentially modulating menstrual physiology and pain perception^19^.

Given these multifaceted mechanisms, probiotic supplementation offers a compelling, non-pharmacologic approach for managing primary dysmenorrhea. Targeting the inflammatory component of the disorder through microbiota-directed therapies may improve symptoms while minimizing the adverse effects associated with pharmacological treatments. This study, therefore, aims to evaluate the impact of oral multispecies probiotic supplementation on menstrual pain severity in young women with primary dysmenorrhea.

## Materials and methods

### Study design

This study was a double-blinded, randomized, placebo-controlled trial conducted in accordance with the CONSORT guidelines. The trial was carried out at the Department of Obstetrics and Gynecology, Faculty of Medicine, Ramathibodi Hospital, Mahidol University, Bangkok, Thailand. The intervention period lasted for three months, followed by a three-month post-intervention observational follow-up.

The primary outcome was the change in dysmenorrhea pain severity measured by the visual analog scale (VAS). The secondary outcomes were to assess (1) the persistence of pain reduction after discontinuation of probiotic supplementation during the three-month follow-up period, (2) changes in menstrual characteristics, including flow volume, and (3) the incidence and nature of adverse events throughout the study period. The study protocol was approved by the Institutional Review Board (COA MURA2023/136, approved on February 27, 2023), and the trial was registered in the Thai Clinical Trials Registry (TCTR20230326001). All procedures performed in this study were in accordance with the ethical standards of the institutional and national research committees and with the 1964 Helsinki Declaration and its later amendments, or with comparable ethical standards.

### Participants

Eligible participants were healthy females aged 18–24 years with regular menstrual cycles (21–35 days) and self-reported moderate to severe primary dysmenorrhea, defined as VAS pain scores > 5 for at least three consecutive cycles. Diagnosis was made based on clinical history and symptom pattern. Pelvic ultrasonography was performed to exclude secondary causes such as endometriosis or structural abnormalities. Exclusion criteria included known or suspected secondary dysmenorrhea, chronic medical conditions, prior pelvic or abdominal surgery, hormonal or supplement use within the past three months, plans for pregnancy, and known allergies to probiotics.

### Recruitment and setting

This was a single-center study conducted at the Department of Obstetrics and Gynecology, Faculty of Medicine, Ramathibodi Hospital, Mahidol University, Bangkok, Thailand. Participants were recruited from the outpatient gynecology clinic at Ramathibodi Hospital. Potentially eligible individuals presenting for consultation were screened by the research team and invited to participate. Enrollment, therefore, represented a convenience sample of eligible volunteers attending the clinic during the recruitment period. Participant recruitment and data collection were conducted from March 30, 2023, to November 30, 2023.

### Sample size calculation

Based on a previous clinical trial evaluating VAS scores in dysmenorrhea^20^, we estimated that 48 participants (24 per group) would provide 80% power to detect a clinically meaningful between-group difference at α = 0.05, allowing for potential attention. Although 48 participants were randomized, 40 participants (20 per group) completed the intervention and contributed outcome data for the primary analysis.

 With 20 participants per group, a two-sample comparison at α = 0.05 retains 80% power to detect a standardized mean difference of approximately Cohen’s d ≈ 0.89, corresponding to a relatively large effect size. In the present study, the observed between-group difference in VAS score at Month 3 (3.7 ± 1.84 vs 5.8 ± 2.14) corresponded to Cohen’s d ≈ 1.05, which exceeds this detectable effect size threshold, suggesting that the final sample size remained sufficient to detect the observed primary-outcome difference.

### Randomization and intervention

After providing written informed consent, eligible participants were randomly assigned in a 1:1 ratio using computer-generated block randomization (block size of four). Allocation was concealed using sealed, opaque, sequentially numbered envelopes prepared by an independent staff member not involved in the study. Both participants and investigators were blinded to group allocation throughout the study. The intervention group received multispecies probiotic sachets containing *inulin (200 mg), Polydextrose (200 mg),and a blend of Lactobacillus and Bifidobacterium species with a labeled potency of* > *2.5* × *10*^*1*^*⁰ CFU per sachet (per day).*Probiotic doses used in human studies vary by strain and indication; however, commonly marketed and clinically studied probiotic products frequently provide doses in the range of approximately 10⁹–10^11^ CFU/day, and dose requirements are recognized to be strain- and outcome-specific. The selected dose in the present trial (> 2.5 × 10^1^⁰ CFU/day), therefore, falls within the range commonly used in clinical research and practice guidelines for probiotic supplementation^21^*.* While placebo sachets were identical in appearance and taste, they contained only inulin (200 mg). Both sachets were manufactured by CMED PRODUCTS 1994 Co., Ltd., Bangkok, Thailand. Participants were instructed to consume one sachet daily and avoid additional probiotic-containing foods during the trial. Use of NSAIDs or paracetamol was allowed for breakthrough pain and recorded in monthly logs.

### Outcome measurements

Baseline demographic data and menstrual history were collected at the time of enrollment. Pain severity was assessed monthly using self-reported VAS scores (0–10 scale), recorded at the peak of menstrual pain during each cycle. Compliance was monitored via monthly follow-ups using the LINE application. Adherence was defined as consuming ≥ 80% of the assigned sachets. After completing the intervention phase, participants continued to record VAS scores for three additional menstrual cycles during the follow-up period. To facilitate clinical interpretation, we prespecified a clinically meaningful improvement in pain intensity as a ≥ 2-point reduction on the 0–10 scale (or ≥ 30% from baseline), consistent with IMMPACT recommendations for interpreting clinically important change in pain intensity outcomes^22^.

Menstrual flow volume was estimated using a menstrual record completed each cycle. Participants recorded the number and type of sanitary pads used per day and the degree of saturation (e.g., lightly, moderately, or fully soaked). These records were converted to an estimated menstrual blood loss (mL) using a pictorial pad-based scoring approach (modified pictorial blood loss assessment method), where higher pad counts and greater saturation correspond to greater estimated volume^23^. The total estimated volume per cycle was calculated by summing daily values.

### Statistical analysis

All data were analyzed using STATA version 16.0 (StataCorp, College Station, TX, USA). Continuous variables were assessed for normality using the Shapiro–Wilk test and compared between groups using Student’s t-test or the Mann–Whitney U test, as appropriate. Categorical variables were analyzed using the chi-squared test or Fisher’s exact test as appropriate. The primary outcome was the change in menstrual pain severity (VAS) over six months. A linear mixed-effects model with repeated measures was used to assess the effects of group, time, and the interaction between group and time as fixed effects, with participant ID as a random effect. This accounted for the within-subject correlation and missing data. The key parameter of interest was the interaction between the group and time All statistical tests were two-tailed, with a significance level set at p < 0.05. Analyses were conducted using a per-protocol (complete-case) approach, including participants who completed the 3-month intervention and provided primary outcome data (n = 40; 20 per group). Participants who were lost to follow-up or discontinued the intervention were excluded from the per-protocol analysis set. Participants were analyzed according to their randomized group assignment among completers. A two-sided p-value < 0.05 was considered statistically significant.

## Results

**Fig. 1:**
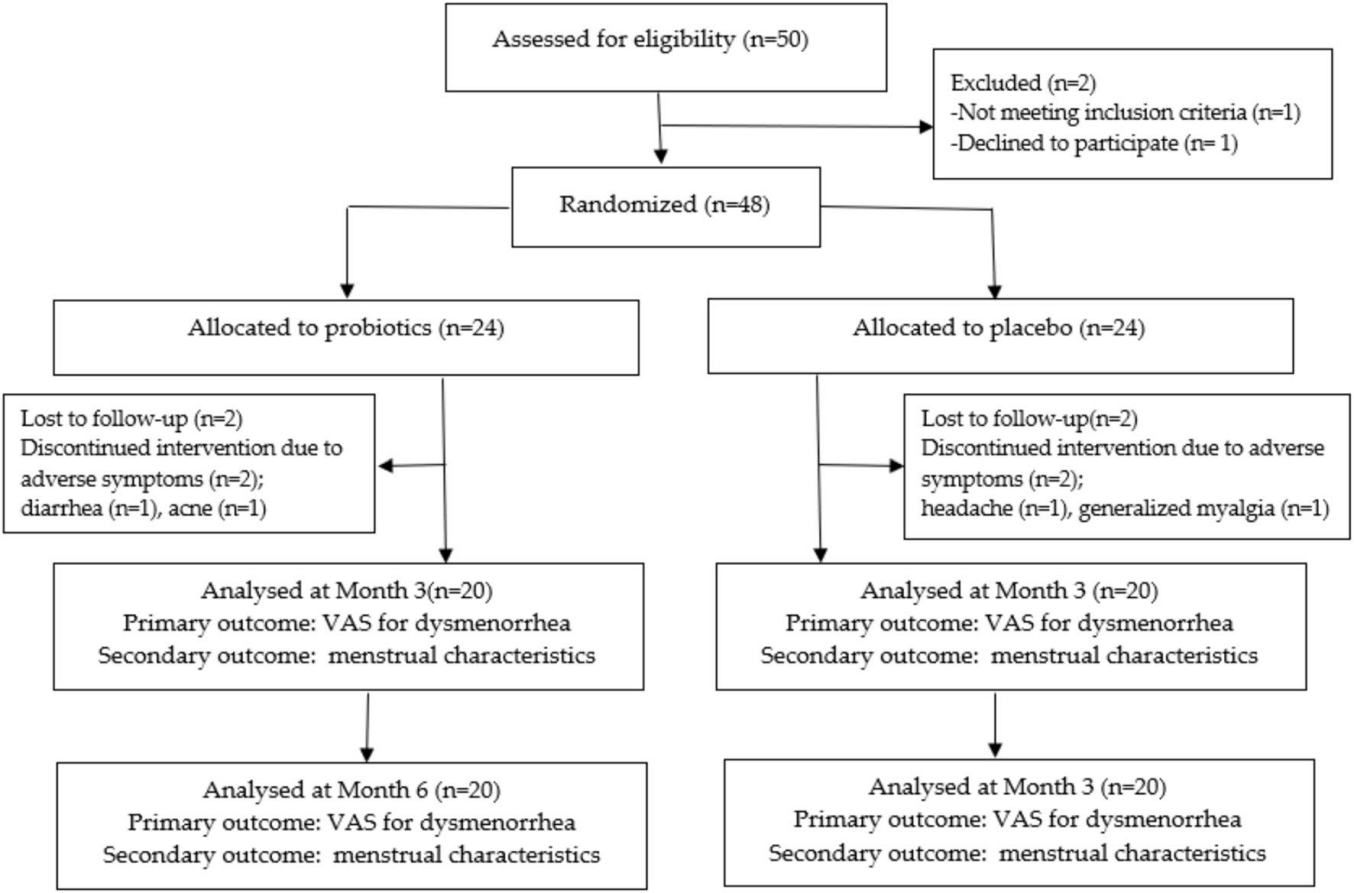
CONSORT flow diagram of participant enrollment, randomization, and follow-up. Fifty individuals were assessed for eligibility; 48 were randomized (probiotic, n = 24; placebo, n = 24). During follow-up, two participants in each group were lost to follow-up and two participants in each group discontinued due to symptoms (probiotic: diarrhea, acne; placebo: headache, generalized myalgia). Forty participants completed the 3-month intervention and were included in the Month 3 analysis (n = 20 per group). The same 40 participants were followed through Month 6 (n = 20 per group). VAS: Visual Analog Scale.

Of the 50 women screened for eligibility, 48 met the inclusion criteria and were randomized equally into two groups: a probiotic group (n = 24) and a placebo group (n = 24) **(**Fig. 1**)**. A total of 40 participants (20 per group) completed the six-month study; four participants in each group were lost to follow-up during the intervention phase. Compliance with the intervention was high, corresponding to 100% adherence in both the probiotic and placebo groups, as confirmed by sachet counts during LINE telecommunication follow-ups. Baseline characteristics, including age, body mass index (BMI), age at menarche, menstrual cycle duration, menstrual flow volume, pre-intervention VAS pain scores, history of sexual intercourse, and exercise duration, showed no significant differences between the groups (P > 0.05) (Table 1).

**Table 1:** Baseline characteristics of the participants.

**Characteristic**	**Probiotics group** **(n = 20)**	**Placebo group (n = 20)**	P-value
Age (years)	21.95 ± 0.94	21.7 ± 0.92	0.403
Weight (Kg)	51.95 ± 4.70	50.43 ± 5.10	0.333
Height (CM)	161.48 ± 3.99	161.05 ± 5.64	0.785
Body mass index(kg/m^2^)	19.94 ± 1.88	19.44 ± 1.70	0.380
Age at menarche (years)*	12 (10,17)	12 (11,14)	0.311
Duration of the menstrual cycle	5.5 ± 1.24	5.15 ± 0.99	0.329
Amount of menstrual cycle (ml)*	50 (25,200)	55 (20,150)	0.752
Pain score before intervention	6.1 ± 1.17	6.3 ± 1.26	0.605
History of sexual intercourse** No	12 (60%)	11 (55%)	0.749
Yes	8 (40%)	9 (45%)	
Exercise time** < 150 min/week	15 (75%)	18 (90%)	0.407
> 150 min/week	5 (25%)	2 (10%)	

Data are presented as mean ± SD, *median (min, max), and **N (%).

### Primary outcome

**Fig. 2:**
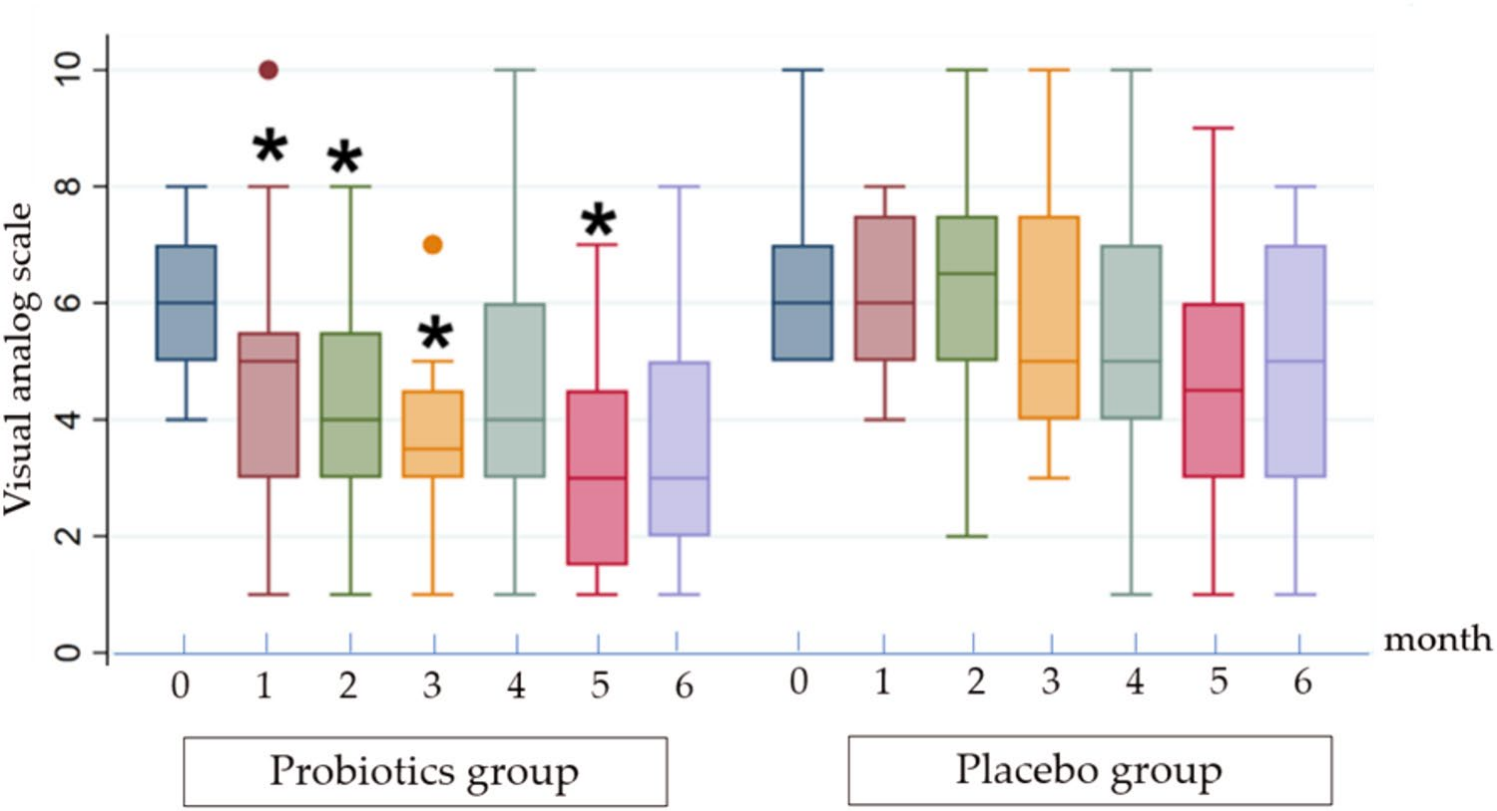
Changes in dysmenorrhea severity over time. box plots of monthly visual analog scale (VAS; 0–10) scores in the probiotic and placebo groups from baseline (Month 0) through Month 6. Asterisks (*) indicate statistically significant between-group differences in VAS scores at the corresponding month (probiotic vs placebo; p < 0.05). Between-group p-values for each month are provided in Table 2. Boxes represent the interquartile range with median lines; whiskers indicate the range.

Mean VAS pain scores were comparable at baseline between the probiotic and placebo groups (6.1 ± 1.17 vs 6.3 ± 1.26; p = 0.62). During the supplementation phase, the probiotic group had significantly lower VAS scores than the placebo group at Month 1 (4.6 ± 2.21 vs 6.25 ± 1.37; p < 0.01), Month 2 (4.35 ± 1.87 vs 6.3 ± 2.11; p < 0.01), and Month 3 (3.7 ± 1.84 vs 5.8 ± 2.14; p < 0.01). Following discontinuation, between-group differences were not consistent: the difference was not statistically significant at Month 4 (4.5 ± 2.26 vs 5.45 ± 2.26; p = 0.19), was significant at Month 5 (3.15 ± 1.79 vs 4.7 ± 2.15; p = 0.02), and was not significant at Month 6 (3.55 ± 1.96 vs 4.85 ± 2.18; p = 0.06). (Table 2**, **Fig. 2**).** Overall, probiotic supplementation was associated with lower VAS scores during active treatment, while post-intervention differences fluctuated and were not consistently maintained across follow-up months.

**Table 2:** Visual analog scale (VAS) dysmenorrhea pain scores over time.

Months of study	Probiotics group (n = 20)	Placebo group (n = 20)	P-value
0	6.1 ± 1.17	6.3 ± 1.26	0.62
1	4.6 ± 2.21	6.25 ± 1.37	< 0.01*
2	4.35 ± 1.87	6.3 ± 2.11	< 0.01*
3	3.7 ± 1.84	5.8 ± 2.14	< 0.01*
4	4.5 ± 2.26	5.45 ± 2.26	0.19
5	3.15 ± 1.79	4.7 ± 2.15	0.02*
6	3.55 ± 1.96	4.85 ± 2.18	0.06

Data are presented as mean ± SD. *Statistically significant P-value < 0.05.

### Secondary outcome and safety

There were no significant differences in menstrual cycle duration, flow volume, or other menstrual characteristics between the two groups throughout the study. Menstrual flow volume, estimated from menstrual records using a pictorial pad-based method, is summarized in Table 3 as median (range). At baseline, estimated menstrual blood loss was comparable between groups (probiotic 50 [25–200] mL vs placebo 55 [20–150] mL; p = 0.752). During the supplementation phase (Months 1–3), no statistically significant between-group differences were observed at any monthly assessment (Month 1: 51.5 [5–180] vs 40 [18–100] mL; p = 0.206; Month 2: 50 [20–160] vs 44 [15–100] mL; p = 0.527; Month 3: 50 [13–168] vs 37 [15–120] mL; p = 0.206). Similarly, during the post-intervention follow-up period (Months 4–6), menstrual flow estimates remained comparable between groups (Month 4: 53.75 [20–100] vs 54 [4–200] mL; p = 1.00; Month 5: 54.5 [12–160] vs 55 [10–120] mL; p = 1.00; Month 6: 62.6 [4.5–160] vs 67.5 [3.5–150] mL; p = 0.752) (Table 3).

**Table 3:** Amount of menstrual volume (ml) between the multispecies probiotics and the placebo group.

Months of study	Probiotics group (n = 20)	Placebo group (n = 20)	P-value
0	50 (25,200)	55 (20,150)	0.752
1	51.5 (5,180)	40 (18,100)	0.206
2	50 (20,160)	44 (15,100)	0.527
3	50 (13,168)	37 (15,120)	0.206
4	53.75 (20,100)	54 (4,200)	1.00
5	54.5 (12,160)	55 (10,120)	1.00
6	62.6 (4.5,160)	67.5 (3.5,150)	0.752

Data shown as median(range) *statistically significant p-value < 0.05.

Reported adverse events included diarrhea, headache, acne, and myalgia. All adverse events were self-limiting, and no serious adverse events occurred. Adverse events were assessed as treatment-emergent symptoms during the study period, and the incidence of experiencing any adverse event was comparable between groups (probiotic: [2/24, 8.33%] vs placebo: [2/24, 8.33%]. Four participants discontinued due to adverse symptoms, including diarrhea and new-onset acne in the probiotic group and headache and generalized myalgia in the placebo group (Fig. 1).

## Discussion

This randomized, double-blind, placebo-controlled trial demonstrated that oral probiotic supplementation significantly reduced menstrual pain severity in young women with primary dysmenorrhea. The effect was evident as early as the first month and sustained throughout the 3-month intervention. However, the therapeutic benefit diminished following discontinuation, suggesting a need for continuous use to maintain symptom relief.

Our findings add to the limited randomized evidence evaluating probiotics for dysmenorrhea and should be interpreted in the context of differences in trial design, probiotic formulation, and outcome selection across studies. In our double-blind, placebo-controlled trial (n = 48 randomized; n = 40 analyzed), a daily multispecies probiotic administered for three menstrual cycles resulted in significantly lower VAS pain scores compared with placebo during the supplementation phase, with less consistent differences after discontinuation. In contrast, the PERIOD study by Zakaria et al^24^. was a larger randomized, double-blind, placebo-controlled trial (n = 72; 36 per arm) conducted in Malaysia that used a multispecies formulation (Lactobacillus and Bifidobacterium strains) administered twice daily for 3 months; that trial did not demonstrate a significant between-group difference in overall quality-of-life scores or inflammatory cytokines, although it reported lower NSAID use and improved mental health scores in the probiotic arm. These differences may reflect variation in primary endpoints (pain intensity versus quality of life), background analgesic use and confounding by NSAIDs, and differences in strain composition and dosing frequency. More recent randomized studies assessing probiotics for menstrual symptoms have also reported heterogeneous effects. More recent randomized trials have likewise shown mixed findings. For example, Yang et al. (2025) conducted a double-blind RCT in women with primary dysmenorrhea (PD n = 58) plus a non-PD reference group (n = 10), and administered a daily multi-strain probiotic capsule containing Bifidobacterium longum subsp. longum OLP-01, Lactobacillus plantarum PL-02, and Lactococcus lactis LY-66 (1:1:1; 1.5 × 10^1^⁰ CFU/day) for 10 weeks, with or without twice-weekly HIIT; improvements were more evident in menstrual distress symptoms, while effects on pain intensity and related biomarkers were not consistently demonstrated^25^.

Mechanistically, primary dysmenorrhea is associated with increased uterine prostaglandins (notably PGF2α and PGE2), which promote myometrial hypercontractility and uterine ischemia, generating cramping pain^26^. Probiotics may plausibly influence dysmenorrhea through immunomodulatory and neuroendocrine pathways, including downregulation of inflammatory mediators such as TNF-α and CRP (as suggested by meta-analytic evidence)^27^ and modulation of gut–brain signaling relevant to pain processing^28,29^. Notably, although pain outcomes improved during supplementation, we did not observe consistent between-group differences in menstrual cycle length or estimated menstrual flow volume, suggesting that the primary impact of probiotics may be mediated through immune/nociceptive modulation rather than alterations in reproductive hormonal regulation, consistent with literature indicating that synbiotic/probiotic supplementation can modify immune parameters and microbiota without clear disruption of hormonal axes^30^.

We observed a gradual reduction in VAS pain scores in the placebo group over successive cycles. Improvement in placebo/control arms is common in pain trials and may reflect contextual (placebo/expectancy) effects, regression to the mean, and the natural fluctuation of symptoms across menstrual cycles^31^. In addition, repeated monthly symptom recording and regular follow-up contact may contribute to measurement reactivity/Hawthorne-type effects, whereby the act of monitoring and being observed can influence behaviors (e.g., optimizing timing of usual analgesics or self-care) and symptom reporting^32,33^. These non-specific effects may partially explain the downward trend in the placebo group and underscore the importance of interpreting changes primarily through between-group comparisons. Our findings indicate that benefits are most evident during active supplementation, suggesting probiotic-related immunomodulatory and gut–brain effects may require ongoing exposure. The less consistent post-discontinuation effects raise the question of whether probiotics produce durable microbiota remodeling or only transient effects. Supporting this, persistence after cessation appears strain- and host-dependent: in a pilot study of 30 healthy adults, Tremblay et al. found that after 2 weeks of a multi-strain probiotic (30 × 10⁹ CFU/day), fecal detection typically persisted only for several days after stopping in many strains, with longer persistence in some individuals^34^. These observations underscore the need for longer-term trials with repeated microbiome profiling and mechanistic biomarkers (e.g., inflammatory markers and prostaglandins) to evaluate the durability of benefit.

Beyond primary dysmenorrhea, probiotics may have broader applications in gynecologic conditions characterized by chronic inflammation, such as endometriosis. Clinical studies have demonstrated that *Lactobacillus gasseri* supplementation significantly reduces pelvic pain in women with endometriosis, supporting the role of probiotics in modulating inflammatory responses in estrogen-sensitive disorders^20,35^. Mechanistically, probiotics may act through multiple pathways, including enhancement of gut microbial diversity, restoration of mucosal barrier function, and reduction of systemic inflammation. Dysbiosis has been linked to increased intestinal permeability and elevated production of pro-inflammatory cytokines, both of which may exacerbate menstrual pain. By restoring microbial equilibrium, probiotics may attenuate these inflammatory pathways and improve pain perception.

Additionally, the gut microbiota has been implicated in estrogen metabolism via the estrobolome (bacterial genes involved in deconjugation and recirculation of estrogens), which may be relevant to estrogen-dependent gynecologic conditions^19,36^. However, our trial did not demonstrate consistent between-group differences in menstrual cycle characteristics or estimated menstrual flow volume, suggesting that the observed improvement in pain during supplementation is more likely mediated through immune and nociceptive modulation (e.g., inflammatory signaling and gut–brain pathways) rather than clinically meaningful alterations in systemic estrogen activity. Accordingly, we interpret the estrobolome as a plausible background mechanism in related disorders, while emphasizing that our findings primarily support probiotics as an adjunct for symptom modulation in primary dysmenorrhea. 

## Strengths & limitations

This randomized, double-blind, placebo-controlled trial offers preliminary clinical evidence supporting the short-term efficacy of probiotic supplementation in reducing menstrual pain among women with primary dysmenorrhea. Key methodological strengths include robust allocation concealment, standardized outcome measurement, and a post-intervention observation period, which allows for the evaluation of both acute and residual treatment effects.

However, several limitations warrant consideration. First, the single-center design and homogeneity of the study population (predominantly Asian women) may limit generalizability. Second, the follow-up duration was limited to three months post-intervention, precluding conclusions about long-term efficacy or sustainability of benefit. Third, the absence of biological or inflammatory markers restricts mechanistic interpretation of the findings.

### Implications for future research and clinical practice

The observed short-term benefit of probiotic supplementation highlights its potential as a safe, accessible, and non-pharmacological option for managing primary dysmenorrhea, particularly for individuals seeking alternative therapies. Importantly, our findings extend the emerging literature by providing prospective, double-blind randomized evidence that a multispecies probiotic regimen can reduce VAS-rated peak menstrual pain over three consecutive cycles in young women with moderate-to-severe primary dysmenorrhea. The variable findings after discontinuation underscore that duration of therapy and maintenance dosing are likely important determinants of clinical response and should be explicitly tested in future trials.

Future studies should aim to validate these findings in larger, ethnically diverse cohorts and incorporate biomarker analyses, including prostaglandin levels, systemic inflammatory markers, and gut microbiota composition. Such studies could elucidate the underlying pathways of probiotic action and support the development of personalized, mechanism-based interventions for dysmenorrhea.

## Conclusion

In this double-blind, randomized, placebo-controlled trial, multispecies probiotics reduced VAS-rated dysmenorrhea pain during active supplementation compared with placebo, with less consistent effects after discontinuation. These results provide controlled-trial evidence supporting probiotics as a non-hormonal adjunct and identify key gaps for future work on the durability of benefit, dosing, and mechanisms.

## Data Availability

The data that support the findings of this study are not openly available due to reasons of sensitivity, but are available from the corresponding author upon reasonable request.

## References

[1] Ferries-Rowe, E., Corey, E. & Archer, J. S. Primary dysmenorrhea: Diagnosis and therapy. *Obstet. Gynecol.* **136**(5), 1047–1058. 10.1097/AOG.0000000000004104 (2020). 33030880 10.1097/AOG.0000000000004096

[2] American College of Obstetricians and Gynecologists. ACOG committee opinion No. 760: Dysmenorrhea and endometriosis in the adolescent. *Obstet. Gynecol.* **132**(6), e249–e258. 10.1097/AOG.0000000000002967 (2018). 30461694 10.1097/AOG.0000000000002978

[3] MacGregor, B. et al. Disease burden of dysmenorrhea: impact on life course potential. *Int J Womens Health.* **15**, 499–509. 10.2147/IJWH.S371234 (2023). 37033122 10.2147/IJWH.S380006 PMC10081671

[4] Sharma, A. et al. Problems related to menstruation and their effect on daily routine of students of a medical college in Delhi, India. *Asia Pac. J. Public Health.* **20**(3), 234–241. 10.1177/1010539508316939 (2008). 19124317 10.1177/1010539508316939

[5] Chuamoor, K., Kaewmanee, K. & Tanmahasamut, P. Dysmenorrhea among Siriraj nurses; prevalence, quality of life, and knowledge of management. *J. Med. Assoc. Thai.* **95**(8), 983–991 (2012). 23061300

[6] Iacovides, S., Avidon, I. & Baker, F. C. What we know about primary dysmenorrhea today: A critical review. *Hum. Reprod. Update.* **21**(6), 762–778. 10.1093/humupd/dmv039 (2015). 26346058 10.1093/humupd/dmv039

[7] Wang, L. et al. Prevalence and risk factors of primary dysmenorrhea in students: a meta-analysis. *Value Health.* **25**(10), 1678–1684. 10.1016/j.jval.2022.06.014 (2022). 35523614 10.1016/j.jval.2022.03.023

[8] Chen, C. X. et al. Reasons women do not seek health care for dysmenorrhea. *J Clin Nurs.* **27**(1–2), e301–e308. 10.1111/jocn.13937 (2018). 28681499 10.1111/jocn.13946 PMC5746430

[9] Lundström, V. & Gréen, K. Endogenous levels of prostaglandin F2alpha and its main metabolites in plasma and endometrium of normal and dysmenorrheic women. *Am. J. Obstet. Gynecol.* **130**(6), 640–646. 10.1016/0002-9378(78)90189-6 (1978). 637076 10.1016/0002-9378(78)90320-4

[10] Ma, H. et al. Altered cytokine gene expression in peripheral blood monocytes across the menstrual cycle in primary dysmenorrhea: A case-control study. *PLoS ONE* **8**(2), e55200. 10.1371/journal.pone.0055200 (2013). 23390521 10.1371/journal.pone.0055200 PMC3563666

[11] Barcikowska, Z. et al. Inflammatory markers in dysmenorrhea and therapeutic options. *Int. J. Environ. Res. Public Health* **17**(4), 1191. 10.3390/ijerph17041191 (2020). 32069859 10.3390/ijerph17041191 PMC7068519

[12] Burnett, M. & Lemyre, M. No. 345–Primary dysmenorrhea consensus guideline. *J. Obstet. Gynaecol. Can.* **39**(7), 585–595. 10.1016/j.jogc.2016.12.023 (2017). 28625286 10.1016/j.jogc.2016.12.023

[13] Yonglitthipagon, P. et al. Effect of yoga on the menstrual pain, physical fitness, and quality of life of young women with primary dysmenorrhea. *J. Bodyw. Mov. Ther.* **21**(4), 840–846. 10.1016/j.jbmt.2017.01.014 (2017). 29037637 10.1016/j.jbmt.2017.01.014

[14] Liu, C. Z. et al. Immediate analgesia effect of single point acupuncture in primary dysmenorrhea: a randomized controlled trial. *Pain Med.* **12**(2), 300–307. 10.1111/j.1526-4637.2010.01046.x (2011). 21166767 10.1111/j.1526-4637.2010.01017.x

[15] Xu, Y., Yang, Q. & Wang, X. Efficacy of herbal medicine (cinnamon/fennel/ginger) for primary dysmenorrhea: A systematic review and meta-analysis of randomized controlled trials. *J. Int. Med. Res.* **48**(6), 300060520936179. 10.1177/0300060520936179 (2020). 32603204 10.1177/0300060520936179 PMC7328489

[16] Hill, C. et al. The International Scientific Association for Probiotics and Prebiotics consensus statement on the scope and appropriate use of the term probiotic. *Nat. Rev. Gastroenterol. Hepatol.* **11**(8), 506–514. 10.1038/nrgastro.2014.66 (2014). 24912386 10.1038/nrgastro.2014.66

[17] Singh, R. K. et al. Influence of diet on the gut microbiome and implications for human health. *J. Transl. Med.* **15**(1), 73. 10.1186/s12967-017-1175-y (2017). 28388917 10.1186/s12967-017-1175-y PMC5385025

[18] Wu, L. Y. et al. The role of probiotics in women’s health: an update narrative review. *Taiwan J Obstet Gynecol.* **63**(1), 29–36. 10.1016/j.tjog.2023.11.002 (2024). 38216265 10.1016/j.tjog.2023.09.018

[19] Baker, J. M., Al-Nakkash, L. & Herbst-Kralovetz, M. M. Estrogen-gut microbiome axis: Physiological and clinical implications. *Maturitas.* **103**, 45–53. 10.1016/j.maturitas.2017.06.025 (2017). 28778332 10.1016/j.maturitas.2017.06.025

[20] Itoh, H. et al. Lactobacillus gasseri OLL2809 is effective especially on the menstrual pain and dysmenorrhea in endometriosis patients: randomized, double-blind, placebo-controlled study. *Cytotechnology* **63**(2), 153–161. 10.1007/s10616-011-9364-4 (2011). 21153437 10.1007/s10616-010-9326-5 PMC3080472

[21] Guarner, F. et al. World Gastroenterology Organisation Global Guidelines: Probiotics and prebiotics. *J. Clin. Gastroenterol.* **58**(6), 533–553. 10.1097/MCG.0000000000002002 (2024). 38885083 10.1097/MCG.0000000000002002

[22] Dworkin, R. H. et al. Interpreting the clinical importance of treatment outcomes in chronic pain clinical trials: IMMPACT recommendations. *J. Pain.* **9**(2), 105–121. 10.1016/j.jpain.2007.09.005 (2008). 18055266 10.1016/j.jpain.2007.09.005

[23] Magnay, J. L., O’Brien, S., Gerlinger, C. & Seitz, C. Pictorial methods to assess heavy menstrual bleeding in research and clinical practice: A systematic literature review. *BMC Womens Health.* **20**(1), 24. 10.1186/s12905-020-0887-y (2020). 32041594 10.1186/s12905-020-0887-y PMC7011238

[24] Zakaria, I. A. et al. The role of probiotics in improving menstrual health in women with primary dysmenorrhoea: A randomized, double-blind, placebo-controlled trial (the PERIOD study). *Women Health.* **20**, 17455057241234524. 10.1177/17455057241234524 (2024). 10.1177/17455057241234524 PMC10916465 38444064

[25] Yang, M. Y., Chen, H. Y., Ho, C. H. & Huang, W. C. Impact of probiotic supplementation and high-intensity interval training on primary dysmenorrhea: A double-blind, randomized controlled trial investigating inflammation and hormonal modulation. *Nutrients* **17**(4), 622. 10.3390/nu17040622 (2025). 40004951 10.3390/nu17040622 PMC11858197

[26] Chan, W. Y. & Hill, J. C. Determination of menstrual prostaglandin levels in non-dysmenorrheic and dysmenorrheic subjects. *Prostaglandins* **15**(2), 365–375. 10.1016/0090-6980(78)90138-3 (1978). 635225 10.1016/0090-6980(78)90176-4

[27] Maia, L. P. et al. Effects of probiotic therapy on serum inflammatory markers: a systematic review and meta-analysis. *J Funct Foods.* **54**, 466–478. 10.1016/j.jff.2019.01.045 (2019).

[28] Lin, B. et al. Gut microbiota regulates neuropathic pain: Potential mechanisms and therapeutic strategy. *J. Headache Pain* **21**(1), 103. 10.1186/s10194-020-01170-x (2020). 32807072 10.1186/s10194-020-01170-x PMC7433133

[29] Crocetta, A. et al. From gut to brain: Unveiling probiotic effects through a neuroimaging perspective—A systematic review of randomized controlled trials. *Front. Nutr.* **11**, 1446854. 10.3389/fnut.2024.1446854 (2024). 39360283 10.3389/fnut.2024.1446854 PMC11444994

[30] Li, X. et al. Effect of synbiotic supplementation on immune parameters and gut microbiota in healthy adults: A double-blind randomized controlled trial. *Gut Microbes* **15**(2), 2247025. 10.1080/19490976.2023.2247025 (2023). 37614109 10.1080/19490976.2023.2247025 PMC10453972

[31] Hafliðadóttir, S. H. et al. Placebo response and effect in randomized clinical trials: Meta-research with focus on contextual effects. *Trials* **22**(1), 493. 10.1186/s13063-021-05454-8 (2021). 34311793 10.1186/s13063-021-05454-8 PMC8314506

[32] Berkhout, C. et al. Defining and evaluating the Hawthorne effect in primary care: A systematic review and meta-analysis. *Front. Med.* **9**, 1033486. 10.3389/fmed.2022.1033486 (2022). 10.3389/fmed.2022.1033486 PMC9679018 36425097

[33] Aaron, L. A., Turner, J. A., Mancl, L., Brister, H. & Sawchuk, C. N. Electronic diary assessment of pain-related variables: Is reactivity a problem?. *J. Pain* **6**(2), 107–115. 10.1016/j.jpain.2004.11.003 (2005). 15694877 10.1016/j.jpain.2004.11.003

[34] Tremblay, A. et al. Total transit time and probiotic persistence in healthy adults: A pilot study. *J. Neurogastroenterol. Motil.* **29**(2), 218–228. 10.5056/jnm22031 (2023). 37019866 10.5056/jnm22031 PMC10083121

[35] Khodaverdi, S. et al. Beneficial effects of oral *Lactobacillus* on pain severity in women suffering from endometriosis: A pilot placebo-controlled randomized clinical trial. *Int. J. Fertil. Steril.* **13**(3), 178–183. 10.22074/ijfs.2019.5584 (2019). 31310070 10.22074/ijfs.2019.5584 PMC6642422

[36] Jiang, I. et al. Intricate connections between the microbiota and endometriosis. *Int. J. Mol. Sci.* **22**(11), 5644. 10.3390/ijms22115644 (2021). 34073257 10.3390/ijms22115644 PMC8198999

